# Deep learning for stage prediction in neuroblastoma using gene expression data

**DOI:** 10.5808/GI.2019.17.3.e30

**Published:** 2019-09-27

**Authors:** Aron Park, Seungyoon Nam

**Affiliations:** 1 Department of Health Sciences and Technology, Gachon Advanced Institute for Health Sciences and Technology, Gachon University, Incheon 21565, Korea; 2 Department of Genome Medicine and Science, College of Medicine, Gachon University, Incheon 21565, Korea; 3 Department of Life Sciences, Gachon University, Seongnam 13120, Korea; 4 Gachon Institute of Genome Medicine and Science, Gachon University Gil Medical Center, Incheon 21565, Korea

**Keywords:** deep learning, gene expression, neuroblastoma

## Abstract

Neuroblastoma is a major cause of cancer death in early childhood, and its timely and correct diagnosis is critical. Gene expression datasets have recently been considered as a powerful tool for cancer diagnosis and subtype classification. However, no attempts have yet been made to apply deep learning using gene expression to neuroblastoma classification, although deep learning has been applied to cancer diagnosis using image data. Taking the International Neuroblastoma Staging System stages as multiple classes, we designed a deep neural network using the gene expression patterns and stages of neuroblastoma patients. Despite a small patient population (n = 280), stage 1 and 4 patients were well distinguished. If it is possible to replicate this approach in a larger population, deep learning could play an important role in neuroblastoma staging.

## Introduction

In addition to careful analyses of clinical symptoms, numerous diagnostic methods have been used to diagnose cancer [1]. In particular, cancer is currently staged using various visual methods, such as radiography, computed tomography, bone scans, and positron emission tomography scans [1].

With the increasing amount of available data from visual images over recent years, numerous diagnostic techniques for cancer have been developed through machine learning methods such as convolutional neural networks (CNNs) [2,3]. Moreover, methods for improving the performance of CNNs are being studied, and many models with effective architectures for classifying images have been developed [4]. In recent years, categorical classification models that predict cancer stages or types of cancer have been constructed on the basis of image data [4,5].

In addition to image data, basic classifications, such as a diagnosis of cancerous versus healthy tissue, can be performed through gene expression data, and models have been developed using traditional machine learning methods. However, AI-based deep neural networks (DNNs) can be developed using classification models with data matrices of continuous values such as expression data. Unlike image data, genomic data can be used as a proxy for the early diagnosis of cancer, meaning that models based on gene expression data can also be useful for identifying or predicting the diagnosis or progression of cancer and for providing timely and appropriate cancer treatment [6].

However, to construct a DNN model, a sufficient dataset is required [7]. Although data can be obtained individually, it is possible to secure a sufficiently large dataset to build a model through the Gene Expression Omnibus (GEO) [8] and The Cancer Genome Atlas (TCGA) [9].

In addition to furnishing genomic data, these sources also provide data indicating patients’ medical status, allowing us to examine the correlations between clinical variables and specific genomic data of interest [8,9].

Neuroblastoma is an extracranial solid tumor that most commonly occurs in childhood [10,11]. The specific traits of neuroblastoma include its early age of onset, a tendency for spontaneous regression of the tumor in infancy, and the high frequency of metastatic disease at diagnosis [10].

Neuroblastoma is staged using the International Neuroblastoma Staging System (INSS) [12]. This system classifies tumors in terms of their appearance upon an analysis of surgical biopsy findings, but this staging system alone cannot help doctors to determine a plan for neuroblastoma treatment, since it is dependent upon surgical biopsy findings and its results are obtained post-surgery [12,13].

However, as increasing amounts of data on neuroblastoma have become available, and suitable genomic data can be obtained from public databases (e.g., GEO and TCGA), it is now possible to explore whether a correlation exists between INSS stages and genomic traits such as the mutation profile or gene expression data [14].

In this study, in order to identify such correlations, we developed a simple DNN model using a data set of neuroblastoma patients including gene expression data and clinical data (i.e., INSS stages). We investigated whether our DNN model with gene expression data could classify the INSS stages.

## Methods

### Dataset and data handling

**Fig. 1.**
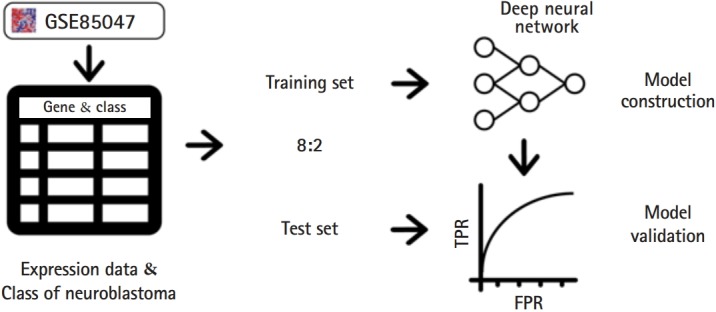
Overview of our model. Our model utilized a simple deep neural network architecture with GSE85047 gene expression data. The classes are the International Neuroblastoma Staging System stage of each patient from GSE85047. False positive rate (FPR), it is the calculated number of predicted false positives divided by the total number of negatives in the test set.; true positive rate (TPR), it is obtained as the number of predicted true positives divided by the total number of positives in the test set.

As a public neuroblastoma dataset, we downloaded accession GSE85047 [15] from the GEO database (https://www.ncbi.nlm.nih.gov/geo/). GSE85047 contains 280 samples of neuroblastoma clinical data and the data matrix includes INSS stage and expression array data. An expression array was performed using an Affymetrix Human Exon 1.0 ST Array (Affymetrix, Santa Clara, CA, USA) (Fig. 1). The INSS stages (1, 2, 3, 4, and 4S) were considered as classes.

In order to convert the array ID into the HUGO gene symbol, we used the GPL5175 probe-gene symbol mapping annotation file. Next, we edited the matrix containing only INSS stage and gene expression array data for data feeding into the DNN architecture. The data matrix was 280 patients by 13,091 gene symbols. We split this data matrix into a training set and test set at a ratio of 8:2, using scikit-learn train_test_split (Fig. 1).

### Model construction and validation

To construct our DNN model, we utilized TensorFlow 1.13.1 as our machine learning library with Python 3.6.0 [16].

**Fig. 2.**
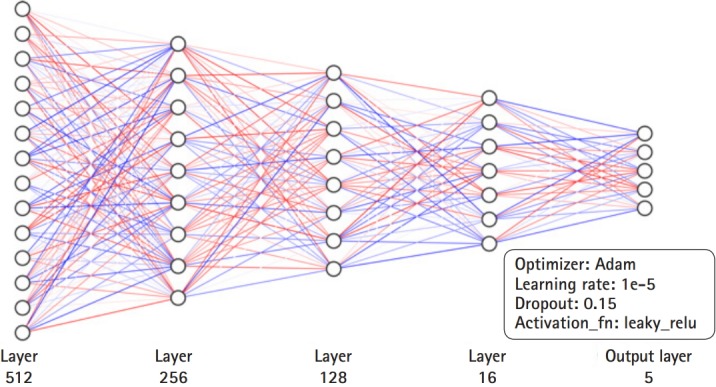
Deep neural network architecture of our model.

We chose tf.contrib.learn.DNNClassifier for model construction. For the hyperparameters of our model, we set the dropout rate at 0.15, we chose the Adam optimizer, and we fixed the learning rate at 1e-5. The activation function was leaky_relu and the number of layers was 4. The numbers of neurons of the layers were 512, 256, 128, and 16, respectively (Fig. 2). When we constructed our model under these hyperparameter settings, we set the number of learning steps as 5,000. An Nvidia Titan RTX 24GB was used for the GPU.

In order to obtain measurements for the performance of our model, accuracy was calculated using the predicted values from the training set and the test set; then, receiver operating characteristic curves and the area under the curve (AUC) were obtained by the roc-curve function in the scikit-learn package.

## Results

After 5,000 iterations with the training set, the accuracy was calculated from each training set and test set, with values of 100% and 55.56%, respectively.

**Fig. 3.**
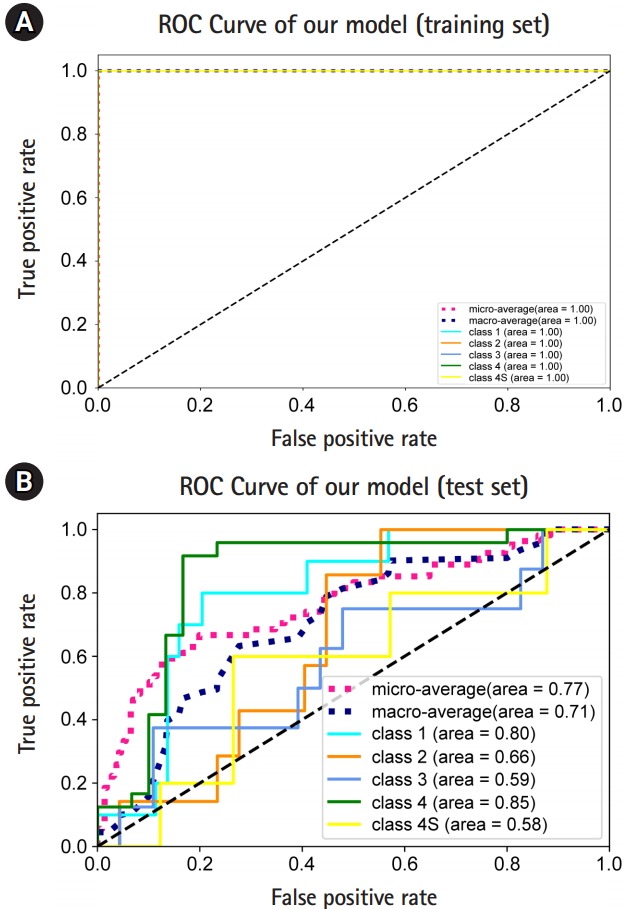
Model performance. (A) Receiver operating characteristic (ROC) curves and the area under the ROCs (AUROCs) of micro-, macro-, and one-versus-rest (OVR) decisions obtained from the training set. (B) The ROC curves and the AUROCs of micro-, macro-, and OVR decisions obtained from the test set.

In the training set, the macro-average AUC, micro-average AUC, and all the AUC values for one-versus-rest (OVR) decisions were all 1 (Fig. 3A). In the test set, we observed a macro-average AUC of 0.71, and a micro-average AUC of 0.77 for five-class classification and prediction using our model. The OVR AUCs for stages (equivalently, classes) 1, 2, 3, 4 and 4S were 0.8, 0.66, 0.59, 0.85, and 0.58, respectively (Fig. 3B). Overall, we observed that our model predicted stage 1 and 4 patients well.

## Discussion

From these results, we could distinguish stages 1 and 4 in neuroblastoma patients. Considering the poor prediction of the other stages in the test set, it is likely that overfitting occurred for stages 2, 3, and 4S. Alternatively, there may be no distinguishable genes between stages 2, 3, and 4S in terms of gene expression.

Our study was performed using data from a relatively small number of patients (280 cases). Increasing the number of patients to the order of 103 or 104 would be appropriate, since successful DNN construction requires several thousand labeled cases in biological problems [7,17].
